# Clinical outcome of patients treated with endoscopic decompression after failure of detorsion for uncomplicated sigmoid volvulus

**DOI:** 10.1002/deo2.299

**Published:** 2023-10-08

**Authors:** Dai Nakamatsu, Tsutomu Nishida, Aya Sugimoto, Kengo Matsumoto, Masashi Yamamoto

**Affiliations:** ^1^ Department of Gastroenterology Toyonaka Municipal Hospital Osaka Japan

**Keywords:** decompression, detorsion, endoscopic treatment, recurrence, sigmoid volvulus

## Abstract

**Background:**

Endoscopic treatment is the first‐line therapy for uncomplicated sigmoid volvulus (SV). However, there are few reports on the clinical course of SV. We investigated the clinical courses of successful and unsuccessful endoscopic detorsions for bowel decompression in patients with uncomplicated SV.

**Methods:**

Between May 2009 and February 2022, patients with uncomplicated SV who underwent endoscopic detorsion or decompression only if detorsion failed were enrolled. A case analysis (all cases) and a patient analysis (first episode cases) were performed. Outcomes were compared between the detorsion and decompression groups, including length of hospital stay, recurrence rate, and days to readmission due to SV.

**Results:**

Seventy patients were included in this study. The success rate of endoscopic detorsion of the SV was 28.6%. There were no differences in age, sex, or other characteristics between the two groups. The hospital stay tended to be longer in the decompression group than in the detorsion group. However, there was no difference in the 30‐day, 6‐month, or 12‐month recurrence rate or the number of days to readmission for SV between the two groups in the case and patient analyses.

**Conclusions:**

This study suggests that endoscopic decompression is a feasible alternative to endoscopic detorsion in patients with uncomplicated SV.

## INTRODUCTION

Sigmoid volvulus (SV) occurs when a loop of the sigmoid colon is twisted along the mesenteric axis, leading to bowel obstruction. Colonic volvulus most commonly occurs in the sigmoid colon and the cecum.^1^ Bowel obstruction occurs when the bowel is rotated 180°, and venous obstruction occurs when there is a 360° rotation. Immediate intervention is necessary to prevent intestinal necrosis or perforation if blood flow is obstructed.^2^ SV accounts for less than 10% of intestinal obstructions in the United States.^3^, ^4^ In Japan, SV accounts for 3.9% of all intestinal obstructions.^5^ However, the incidence of SV varies by region, with a report from Bolivia indicating that SV occurs in 79% of bowel obstruction cases^6^; SV is more common in Africa and the Middle West. The etiological factors of SV include sigmoid colon hypertrophy, abnormal mesenteric fixation, a history of laparotomy, and chronic constipation.^7^ Elderly patients suffering from constipation and patients with neuropsychiatric disorders have reduced mobility of bowel movement and therefore have a high risk of developing SV.^4^, ^8^ As the proportion of aging adults increases, the number of patients with SV is expected to increase.^1^

Endoscopic treatment, including detorsion and decompression, is the first‐line therapy for uncomplicated SV^7^ because it is less invasive. However, the recurrence rate is high even after successful detorsion. Furthermore, cases of SV do not always respond to endoscopic detorsion, and there are limited reports on the differences in the clinical outcomes between successful and unsuccessful detorsion. In this study, we aimed to investigate the clinical outcomes of endoscopic detorsion for bowel decompression in patients with uncomplicated SV, regardless of success.

## MATERIALS AND METHODS

### Study design and patients

This was a retrospective cohort study conducted at a single center. Data on patients with uncomplicated SV were extracted from the endoscopy database of Toyonaka Municipal Hospital between May 2009 and February 2022. Data were retrieved from the electronic medical records using the MegaOak online imaging system (NEC). This study was conducted in accordance with the Declaration of Helsinki and approved by the Institutional Review Board of Toyonaka Municipal Hospital (No. 2022‐08‐05). The requirement of informed consent was waived by opting out on the hospital's website.

During the study period, the eligible patients for this study were divided into two groups: a detorsion group for successful cases of endoscopic detorsion and a decompression group for unsuccessful cases. Additionally, we compared the two groups in all cases (case analysis) and in first episode cases (patient analysis).

### Endoscopic SV detorsion procedure

The endoscopic procedure was initiated following electrocardiogram and SpO_2_ monitoring. The use of sedatives was determined by the patient's condition or comorbidity. We routinely attempted endoscopic detorsion under fluoroscopy using CF‐Q260AI or CF‐H290I (Olympus Optical Co.). CO_2_ insufflation was used in all cases. Emergency endoscopic detorsion was performed by a two‐person team of our trainee and supervising physician. Endoscopic procedures were performed with the goal of successful detorsion in all cases. The number of endoscopic detorsion attempts under fluoroscopy and the decision to abandon detorsion was left to the endoscopist's judgment. In both successful and unsuccessful cases of endoscopic detorsion, the endoscopist confirmed improved bowel dilatation on fluoroscopy at the end of the procedure.

### Diagnosis of SV and definition of endoscopic detorsion

The computed tomography diagnostic criteria for SV are two stenoses caused by twisting of the sigmoid colon and the presence of a dilated intestinal tract with a closed loop between the stenoses. Uncomplicated SV was defined as a condition without evidence of peritonitis, ischemic findings, or perforation.

Successful endoscopic detorsion was defined as passing through two points at the intestinal torsion site, complete endoscopic shortening and straightening of the sigmoid colon, and successful decompression by aspiration of the intestinal gas, leading to subsequent improvement of symptoms.

Decompression was defined as the endoscope being able to pass through the twist point on the anorectal side and being able to be inserted into the dilated sigmoid colon, but detorsion could not be completed. In those cases, the air in the dilated sigmoid colon was suctioned as much as possible to achieve symptomatic improvement.

Retry subsequent detorsion within 2 days without recurrence of abdominal symptoms was included in the detorsion group in this study, regardless of the failure of the first attempt at detorsion. We have termed this method “two‐stage endoscopic detorsion”. Patients for whom detorsion was finally successful were classified as the detorsion group, and the day of the completion of successful detorsion was defined as Day 0. Patients for whom only decompression was achieved and detorsion could not be completed were classified as the decompression group.

Recurrence was defined as obvious recurrent symptoms and computed tomography or X‐ray findings suggestive of SV, such as the mesenteric whirl sign, after improvement by detorsion or decompression.

### Outcome

In the present study, we compared the patients’ background and laboratory data at admission, the clinical course including the length of hospital stay, and the recurrence rate of SV in the detorsion and decompression groups. Regarding the recurrence of SV, we evaluated the total rate of SV recurrence, days to readmission due to SV, and recurrence rate within 30 days, 180 days, and 1 year in the case and patient analyses.

### Statistical analysis

Statistical analyses were performed using JMP statistical software (ver. 16; SAS Institute Inc.). Quantitative variables are presented as the mean ± standard deviation for age and body temperature and as the median and interquartile range for laboratory data on admission, length of hospital stay, and days to readmission. Categorical variables are expressed as frequencies and percentages. The recurrence‐free probability was estimated using the Kaplan‒Meier method and compared using the log‐rank test. Differences in variables were evaluated for statistical significance using t‐tests for quantitative variables and Fisher's exact tests for categorical variables. In the statistical evaluation between the two groups, a *p*‐value <0.05 was considered statistically significant, and *p*‐values were two‐sided. In addition to a *p*‐value, a standardized mean difference was used to compare background characteristics.

## RESULTS

**FIGURE 1:**
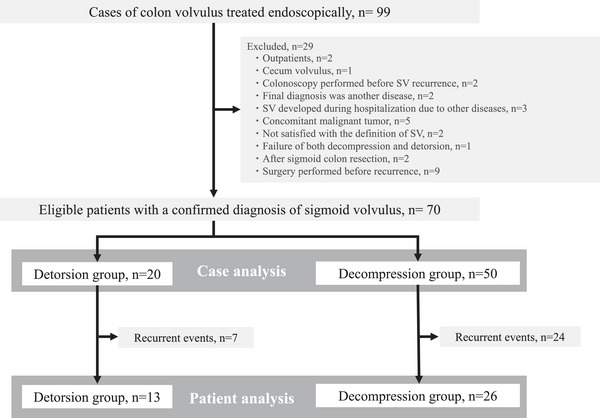
Patient enrollment flow chart for this sigmoid volvulus study.

Ninety‐nine patients with recurrence underwent endoscopic treatment for suspected SV. After excluding 29 patients (nine who underwent surgery for SV without recurrence after the previous event, five with malignant tumors, and 15 with other causes), 70 patients who had endoscopic detorsion, which was attempted to resolve the SV, were included for the case and patient analysis in this study (Figure 1).

A total of 70 enrolled patients were divided into two groups: the detorsion group (*n* = 20), in which endoscopic detorsion was successful, and the decompression group (*n* = 50), in which endoscopic detorsion was unsuccessful and decompression was performed during the same procedure. These two groups were compared for case analysis.

Only 39 patients who suffered from SV for the first time during the study period, excluding the 31 recurrent cases (detorsion group, seven cases, and decompression group, 24 cases), were included in the patient analysis. These patients were divided into the detorsion (*n* = 13) group and the decompression (*n* = 26) group and a comparison was made between these two groups for patient analysis.

Two‐stage endoscopic detorsion was performed in 4 patients in the patient analysis and in nine cases in the case analysis.

The success rate of endoscopic detorsion for SV in this study was 28.6% (20/70) in the case analysis. Table 1 shows the details of the patients’ backgrounds. Age, sex, and commonly found comorbidities in patients with SV, including neuropsychiatric disorders (cerebral hemorrhage, cerebral infarction, psychiatric disorder, Parkinson's disease), constipation, cardiovascular disease, diabetes, and history of abdominal surgery, were evaluated; these characteristics were compared between the detorsion and decompression groups in both the case and patient analyses.

**TABLE 1:** Baseline patient characteristics of the detorsion group and the decompression group (case and patient analyses, respectively).

	Case analysis				Patient analysis			
	Detorsion group	Decompression group	*p*‐value	SMD	Detorsion group	Decompression group	*p*‐value	SMD
Case number	20	50			13	26		
Age (years), mean ± SD	76.4 ± 10.6	74.6 ± 10.9	0.5356	0.17	74.5 ± 11.6	75.8 ± 9.9	0.7470	0.12
Sex, male, *n* (%)	13 (65.0)	33 (66.0)	0.9365	0.02	10 (76.9)	16 (61.5)	0.3367	0.34
Comorbidity, *n* (%)								
Neuropsychiatric disorders	11 (55.0)	33 (66.0)	0.3895	0.23	7 (53.9)	17 (65.4)	0.4850	0.24
Cerebral hemorrhage	0 (0)	3 (6.0)	0.2628	0.36	0 (0)	2 (7.7)	0.3046	0.41
Cerebral infarction	7 (35.0)	12 (24.0)	0.3498	0.24	6 (46.2)	5 (19.2)	0.0782	0.60
Psychiatric disorder	3 (15.0)	7 (14.0)	0.9140	0.03	0	3 (11.5)	0.2024	0.51
Parkinson's disease	0	7 (14.0)	0.0778	0.57	0	4 (15.4)	0.1355	0.60
Others	1 (5.0)	4 (8.0)	0.6597	0.12	1 (7.7)	3 (11.5)	0.7090	0.13
Constipation	14 (70.0)	32 (65.3)	0.7075	0.10	8 (61.5)	16 (64.0)	0.8814	0.05
Cardiovascular disease	4 (20.0)	11 (22.5)	0.8229	0.06	2 (15.4)	8 (32.0)	0.2698	0.40
Diabetes	3 (15.0)	6 (12.2)	0.7579	0.08	3 (23.1)	4 (16.0)	0.5934	0.18
Prior abdominal surgery	5 (25.0)	11 (22.5)	0.8198	0.06	4 (30.8)	6 (24.0)	0.6530	0.15
Present condition at admission								
Body temperature (°C), ± SD	36.7 ± 0.5	36.8 ± 0.5	0.6126	0.19	36.7 ± 0.5	36.9 ± 0.5	0.4756	0.20
Symptom, *n* (%)								
Abdominal pain	10 (50.0)	41 (82.0)	0.0065	0.72	6 (46.2)	19 (73.1)	0.0985	0.57
Abdominal tenderness	9 (45.0)	26 (52.0)	0.5967	0.14	5 (38.5)	11 (42.3)	0.8179	0.08
Nausea	7 (35.0)	9 (18.0)	0.1260	0.39	6 (46.2)	6 (23.1)	0.1410	0.50
Abdominal distension	17 (85.0)	46 (92.0)	0.3778	0.22	12 (92.3)	24 (92.3)	1.0000	0
Laboratory data at admission, median (IQR)								
WBC (/μL)	8200 (6800–11200)	7300 (5900–9000)	0.0971	0.49	8100 (6550–10,800)	8250 (6100–8975)	0.3159	0.37
CRP (mg/dL)	0.47 (0.21–1.75)	0.23 (0.07–0.95)	0.7563	0.07	0.54 (0.22–2.31)	0.42 (0.11–1.41)	0.9325	0.03
CK (mg/dL)	132.5 (72.8–233.3)	77 (55–128)	0.0787	0.53	163.5 (77–304.5)	75 (56.5–103)	0.0729	0.76
LDH (U/L)	218 (205–256.25)	206 (190–245)	0.5340	0.16	226 (207.3–262.8)	223 (186.8–269)	0.7821	0.09

Abbreviations: CK, creatine kinase; CRP, C‐reactive protein; IQR, interquartile range; LDH, lactate dehydrogenase; SD, standard deviation; SMD, standardized mean difference, WBC, white blood cells.

In the case analysis, abdominal pain at the time of admission was significantly more common in the decompression group than in the detorsion group (*p* = 0.0065). There was no difference in the incidence of tenderness, nausea, or abdominal distention at admission or the laboratory data between the detorsion and decompression groups in both the case and patient analyses.

In the patient analysis, the hospital stay tended to be longer in the decompression group than in the detorsion group (10.5 vs. 8 days, *p* = 0.0881), but the difference was not significant. The recurrence rate was similar between the two groups in that the recurrence rates were in the 50%–65% range for case analysis and patient analysis, respectively, with no difference between the detorsion and decompression groups. No complications, such as perforation, were observed in either the detorsion or decompression groups. (Table 2).

**TABLE 2:** Analysis of length of hospital stay and sigmoid volvulus (SV) recurrence in the detorsion group and the decompression group (case and patient analyses, respectively).

	Case analysis			Patient analysis		
	Detorsion group	Decompression group	*p*‐value	Detorsion group	Decompression group	*p*‐value
Number	20	50		13	26	
Length of hospital stay (day), median (IQR)	9 (7–14.8)	11 (8–13)	0.1860	8 (6–11)	11 (9–13)	0.0881
Complications						
Perforation, *n* (%)	0 (0)	0 (0)	1.0000	0 (0)	0 (0)	1.0000
Recurrence after discharge, *n* (%)	13 (65.0)	30 (60.0)	0.6978	8 (61.5)	13 (50.0)	0.4956
95% CI	0.43–0.82	0.46–0.72		0.36–0.82	0.32–0.68	

Abbreviations: CI, confidence interval; IQR, interquartile range.

**FIGURE 2:**
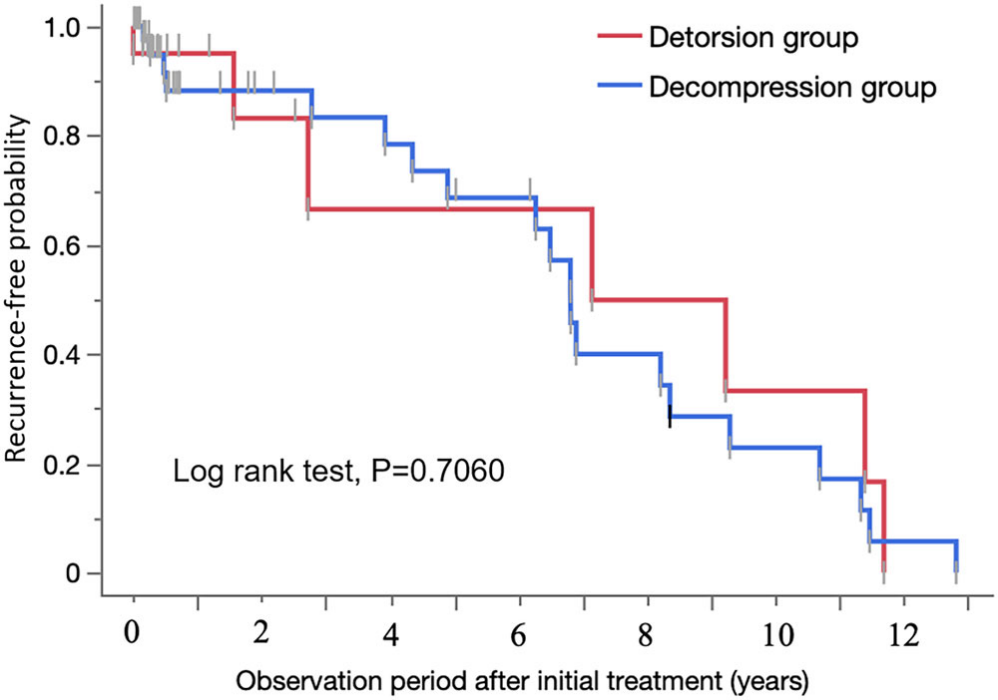
Recurrence‐free probability of patients in the detorsion and decompression groups (case analysis).

**FIGURE 3:**
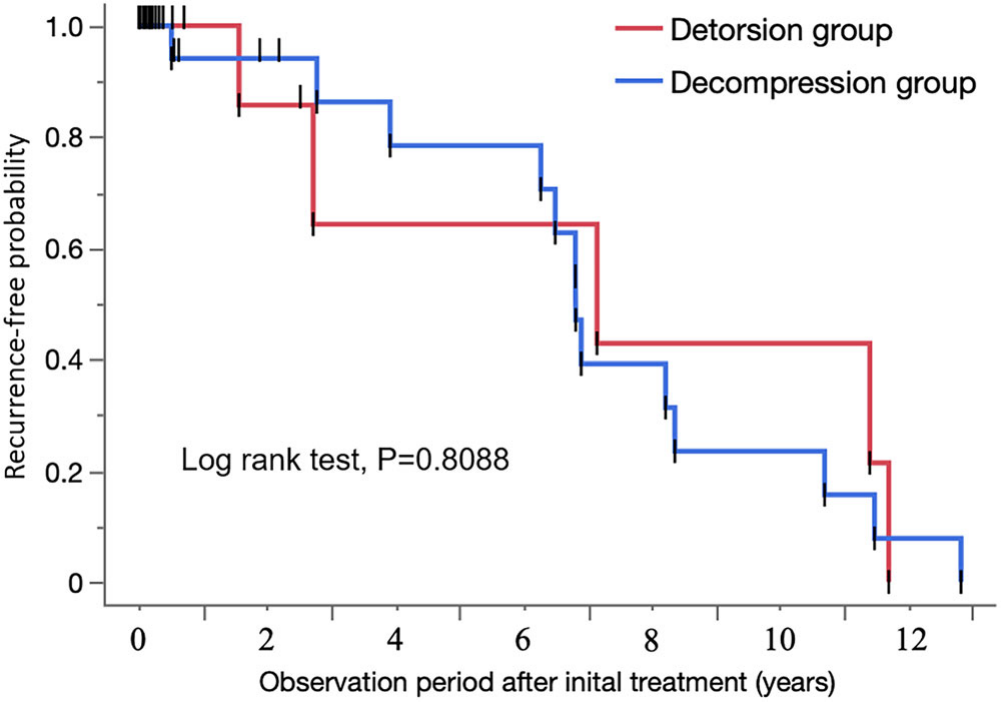
Recurrence‐free probability of patients in the detorsion and decompression groups (patient analysis).

There was no difference in the days to readmission for SV between the detorsion and decompression groups in the case analysis (103 vs. 116 days, *p* = 0.8112) or in the patient analysis (166 vs. 62 days, *p* = 0.3413). There was no difference in recurrence rates within 30 days, 180 days, or 1 year between the two groups (Table 3). The recurrence‐free probabilities between the two groups were also compared by the log‐rank test, but no difference was found in either the case analysis (*p* = 0.7060; Figure 2) or the patient analysis (*p* = 0.8088; Figure 3).

**TABLE 3:** Analysis of readmission due to sigmoid volvulus recurrence in the detorsion group and the decompression group (case and patient analyses, respectively).

	Case analysis			Patient analysis		
	Detorsion group	Decompression group	*p*‐value	Detorsion group	Decompression group	*p*‐value
Number of readmissions, *n* (%)	13 (65.0)	30 (60.0)		8 (61.5)	13 (50.0)	
Days to readmission (day), median (IQR)	103 (83–346)	116 (31.8–248)	0.8112	166 (86–755)	62 (10.5–215)	0.3413
Within 30 days, *n* (%)	5 (38.5)	7 (23.3)	0.3098	1 (12.5)	4 (30.8)	0.3398
Within 180 days, *n* (%)	8 (61.5)	20(66.7)	0.7459	4 (50.0)	9 (69.2)	0.3782
Within 1 year, *n* (%)	10 (76.9)	25 (83.3)	0.6198	6 (75.0)	11 (84.6)	0.5858

Abbreviation: IQR, interquartile range.

## DISCUSSION

Endoscopic decompression and detorsion of the SV have been proposed as the first steps of treatment in the absence of signs of bowel ischemia and perforation.^9^, ^10^ Endoscopic detorsion is considered an ideal approach for patients with uncomplicated SV compared to endoscopic decompression because it has the potential to achieve higher control and a lower recurrence rate. The difficulty of endoscopic detorsion for SV may vary depending on the severity of the condition and any complications that may occur. In addition, there was little evidence of the clinical outcome after the failure of endoscopic detorsion, and only endoscopic decompression was performed. In this retrospective study, we found that endoscopic decompression for SV was a feasible strategy following failed detorsion in a Japanese population. Furthermore, this study revealed the clinical outcome of patients with uncomplicated SV and provided information on successful and unsuccessful endoscopic detorsions for bowel decompression, showing that there were no significant differences between them.

Because untreated SV can cause intestinal strangulation, gangrene, perforation, and peritonitis,^9^, ^10^ treatment including endoscopic detorsion, which has been reported not to increase the risk of complications, is considered useful.^10^, ^11^ On the other hand, endoscopic detorsion for SV can cause intestinal ischemia, necrosis, and perforation, although the risk is not high.^12^ The risk of complications depends on individual cases and factors. In the present study, although we did not observe any severe complications following endoscopy, it is expected that the general condition of SV patients is worse than usual at the onset of SV due to their many comorbidities. ASA (American Society of Anesthesiologists)‐PS (Physical Status) >3 has been reported as an independent risk factor for complications and mortality of SV patients in the 30 days after admission.^13^ Although the length of hospital stay tended to be slightly longer in the decompression group, there was no difference in the recurrence rate of SV or days to readmission between the detorsion group and the decompression group in this study. Therefore, we consider that if a prolonged procedure is judged to be high risk, it may be acceptable to finish with only decompression in a short time, without regard to endoscopic detorsion. The success rate of endoscopic detorsion of SV has been reported to range from 61.9%^5^ to 90%,^10^ and one study reported a success rate of 96% with no complications.^14^ In our study, the success rate of endoscopic detorsion of the SV was 28.6%, which was lower than that reported in previous studies, suggesting that the success rate may depend on factors such as the severity of the condition and the presence of complications. In addition, we defined detorsion as the straightening of the sigmoid colon and release of colon gas under fluoroscopic guidance during endoscopic treatment, and setting a standardized definition may result in a lower success rate of endoscopic detorsion. Furthermore, even after successful detorsion, the recurrence rate (46.2‐60.9%) is reportedly high,^5^, ^13^, ^15^, ^16^ and the recurrence rate of SV in our study was similar to that of previous studies. There was no difference in the subsequent clinical outcome or recurrence rate regardless of the success or failure of endoscopic detorsion, suggesting that decompression alone may be an alternative treatment to detorsion. Therefore, for patients who are concerned about the risk of complications due to the lengthy endoscopic procedure, decompression alone is a better means to finish the procedure early if detorsion is difficult. We believe that endoscopic detorsion has a lower recurrence rate than decompression alone and prolongs the time to SV recurrence. Therefore, in the present study, we observed cases in which 2‐stage endoscopic detorsion was performed a few days after decompression, regardless of the presence or absence of signs of recurrence. However, based on the present results, it may not be necessary to try endoscopic detorsion more than once if the procedure is completed with only decompression.

We speculate that one reason the clinical outcome and recurrence rate did not differ from that of detorsion even when decompression was performed alone may be because colonoscopy was performed for SV treatment under carbon dioxide (CO_2_) insufflation. In the case of air insufflation, once the air enters the oral side of the stenosis, it remains there unless the torsion is released, thereby further aggravating the symptoms. However, as reported by Inoki et al.,^17^ when CO_2_ replaces the air on the oral side of the stenosis, it is promptly absorbed from the intestinal mucosa into the blood and excreted in the exhaled air, which means that the intestinal tract on the oral side can be further reduced after insufflation. We believe that this is one of the reasons why decompression and CO_2_ gas replacement are almost as effective as detorsion. CO_2_ insufflation also has a risk of perforation, and water immersion^18^ or gel immersion^19^ have been mentioned as replacement strategies. In any case, insufflation with CO_2_ is safer than with air, but even with CO_2_, care must be taken to avoid excessive insufflation.

The current study has several limitations. First, this was a single‐center retrospective study, which may limit the generalizability of the findings. Second, the sample size of this study was relatively small, and further research with larger samples would be beneficial to confirm these results. Third, the number of attempts at detorsion is at the discretion of the performing physician, which may lead to differences in the success rate of detorsion and the subsequent clinical course. Fourth, the standardized mean differences exceeded 0.2 for numerous variables, including an imbalance in background characteristics between the case and patient analysis. Although propensity score methods could address this issue, our sample size was insufficient for their effective application. Therefore, we considered that future research should prioritize larger sample sizes and utilize propensity score matching to achieve balanced backgrounds and characteristics. Finally, potential confounding factors were not considered, such as patient comorbidities or other treatments received prior to endoscopic treatment for SV, which could have impacted the outcomes.

In conclusion, this study suggests that endoscopic decompression is a feasible alternative to endoscopic detorsion for bowel degassing in patients with uncomplicated SV.

## CONFLICT OF INTEREST STATEMENT

None.
